# Comparison and clinical utility evaluation of four multiple allergen simultaneous tests including two newly introduced fully automated analyzers

**DOI:** 10.1016/j.plabm.2016.01.002

**Published:** 2016-01-21

**Authors:** John Hoon Rim, Borae G. Park, Jeong-Ho Kim, Hyon-Suk Kim

**Affiliations:** a Department of Laboratory Medicine, Severance Hospital, Yonsei University College of Medicine, Seoul, Republic of Korea; b Department of Medicine, Yonsei University Graduate School of Medicine, Seoul, Republic of Korea

**Keywords:** Multiple allergen simultaneous test, Automated analyzer

## Abstract

**Background:**

We compared the diagnostic performances of two newly introduced fully automated multiple allergen simultaneous tests (MAST) analyzers with two conventional MAST assays.

**Methods:**

The serum samples from a total of 53 and 104 patients were tested for food panels and inhalant panels, respectively, in four analyzers including AdvanSure AlloScreen (LG Life Science, Korea), AdvanSure Allostation Smart II (LG Life Science), PROTIA Allergy-Q (ProteomeTech, Korea), and RIDA Allergy Screen (R-Biopharm, Germany). We compared not only the total agreement percentages but also positive propensities among four analyzers.

**Results:**

Evaluation of AdvanSure Allostation Smart II as upgraded version of AdvanSure AlloScreen revealed good concordance with total agreement percentages of 93.0% and 92.2% in food and inhalant panel, respectively. Comparisons of AdvanSure Allostation Smart II or PROTIA Allergy-Q with RIDA Allergy Screen also showed good concordance performance with positive propensities of two new analyzers for common allergens (*Dermatophagoides farina* and *Dermatophagoides pteronyssinus*). The changes of cut-off level resulted in various total agreement percentage fluctuations among allergens by different analyzers, although current cut-off level of class 2 appeared to be generally suitable.

**Conclusions:**

AdvanSure Allostation Smart II and PROTIA Allergy-Q presented favorable agreement performances with RIDA Allergy Screen, although positive propensities were noticed in common allergens.

## Highlights

• Two new multiple allergen simultaneous tests (MAST) analyzers are evaluated.
• AdvanSure Smart II and PROTIA presented favorable agreement performance with RIDA.
• Positive propensity of new assays for *D. farina* and *D. pteronyssinus* was noticed.
• The changes of cut-off level resulted in total agreement percentage fluctuations.

## 1 Introduction

The detection of allergen-specific IgE, along with the patient’s chief complaints and medical history, is diagnostically valuable for allergic diseases, such as allergic rhinitis, atopic dermatitis, and asthma [1], [2]. Although in vivo skin test has been traditionally used in the clinical environments, there are several limitations of in vivo skin test including error-prone results in patients with anti-histamine medication or skin diseases such as dermographism, possibility of subjective interpretation, and the lack of standardization for protocols [3], [4]. Therefore, in vitro allergen-specific IgE measurements have been developed using various principles of radioimmunoassay, enzyme immunoassay, fluorescent enzyme immunoassay, immunoblot, and chemiluminescent assay [5], [6], [7].

Among the commercially available in vitro allergy tests, multiple allergen simultaneous tests (MAST) have been continuously developed with the improvements in smaller amounts of serum consumption, shorter turnaround time, and wider spectrum of allergens included in the test [6], [8], [9], [10], [11], [12]. Since the difference in prevalence of allergic diseases according to age, sex, and ethnicity is prominent, the selection of multiple allergen screening panels should be modified in the context of geographical regions and race of the target populations [13], [14], [15]. At the same time, the change of environmental substances in modern society must be considered for the progressive development of MAST assays [16].

Moreover, there is no appropriate medical evidence to define any assay as the standardized reference method due to variability of allergen original materials, extraction methods, attachment processes, and detection techniques [5]. Therefore, it is very difficult to analyze true sensitivity, specificity, positive predictive value, and negative predictive value of a specific MAST assay. Nevertheless, actual comparison of new MAST assay with currently used MAST assays can appropriately provide important information in the practical clinical settings.

Recently, two fully automated analyzers with high-throughput were developed and introduced in the market; AdvanSure Allostation Smart II which is the upgraded version of previous AdvanSure AlloScreen by LG Life Science, and PROTIA Allergy-Q which was newly developed by ProteomeTech. Herein, we compared the diagnostic performances of these assays with two most commonly used MAST assays in Korea, today. In addition, we evaluated propensity of each assay to give positive results for certain allergen, which we defined as “positive propensity”.

## 2 Methods

### 2.1 Study participants

We randomly selected the study samples from MAST assay requested serum samples of patients who visited Severance Hospital with symptoms of allergy including urticaria, sneezing, and itching for diagnosis of allergic disease in all age ranges. Additionally, we excluded patients with chronic comorbid diseases such as autoimmunity, malignancy, chronic infection, and other immune-related diseases. Since two different panels were evaluated, we classified patients into two groups so that appropriate panel could be tested based on clinical symptoms and medical records. Due to the variety of allergen types included in the panel of four assays and lack of sufficient sample volume in some patients, different samples were analyzed by different numbers of analyzers with various combinations of allergens. Therefore, only pairs of matched allergens by the same sample were compared among four analyzers.

### 2.2 In vitro allergen-specific IgE measurements

Serum aliquots were tested by four different systems; AdvanSure AlloScreen (LG Life Science, Seoul, Korea), AdvanSure Allostation Smart II (LG Life Science, Seoul, Korea), PROTIA Allergy-Q (ProteomeTech, Seoul, Korea), and RIDA Allergy Screen (R-Biopharm, Darmstadt, Germany). All the test procedures were performed following the manufacture’s instruction. Although detection ranges were various among four analyzers, results were identically classified into 7 levels and were interpreted as class 0–6 in all analyzers (Table 1).

**Table 1:** Specifications of four different MAST analyzers.

	AdvanSure AlloScreen	AdvanSure Allostation Smart II	PROTIA Allergy-Q	RIDA Allergy Screen
Manufacturer	LG Life Science (Korea)	LG Life Science (Korea)	ProteomeTech (Korea)	R-Biopharm (Germany)
Instrument	AdvanSure™ Allostation	AdvanSure™ Allostation Smart II	Q-station	AlleRoboT
Reagent	AdvanSure™ Alloscreen	AdvanSure™ Alloscreen	PROTIA™ Allergy-Q	AlleisaScreen®
Principle	Immunoblot	Immunoblot	Immunoblot	Immunoblot
Class stratification	Class 0–6	Class 0–6	Class 0–6	Class 0–6
Degree of automation	Semi automation	Full automation	Full automation	Full automation
Number of antigens				
-Total (common)	60 (20)	90 (30)	70 (18)	80 (40)
-Food panel	40	60	44	60
-Inhalant panel	40	60	44	60
Minimal sample volume (μl)	100	250	120	800
Tested sample volume (μl)	100	100	50	300
Number of strips utilized	2	2	1	2
Capacity or number of tests per run	24	30	48	36
Analysis time (hr)	3.5	4.0	4.0	3.8
Analysis time per sample (min)	8.75	8	5	6.3

### 2.3 Inter-method comparison of four analyzers

We compared a pair of analyzers each time in order to maximize the comparison efficiency because different allergen lists are available by four analyzers. Furthermore, we focused on comparison of two specific analyzers (AdvanSure AlloScreen versus AdvanSure Allostation Smart II), because AdvanSure Allostation Smart II is the upgraded version of AdvanSure AlloScreen, both of which are developed by the same manufacturer (LG Life Science). Afterwards, we compared two newly introduced analyzers (i.e. AdvanSure Allostation Smart II and PROTIA Allergy-Q) with currently widely utilized assay (RIDA Allergy Screen) as reference values.

### 2.4 Comparison among different cut-off levels for positive interpretation

No standardized specific cut-off level for positive result is defined worldwide until today [17]. Moreover, previous studies which compared various MAST assays utilized different cutoff levels. For instance, several studies used class 1 as the cutoff level for positive results [8], [11], [12], whereas class 2 was adopted as the cutoff level for positive results in other studies [6], [10], [32]. Considering the natural characteristics of semi-quantitative results in MAST assays, comparison of different cut-off levels in the paired results might provide clinical clues for more precise diagnostic interpretation. Therefore, we applied cut-off levels of class 1, class 2, and class 3 as minimal requirement for positive results for all comparison analyses.

### 2.5 Statistical analysis

We analyzed the concordance degree by calculating total agreement percentage following the same methodology used in a previous study [18]; total agreement percentage=(total number of results−number of discrepancies)×100/total number of results. Additionally, concordant positive rates were calculated with the proportions of agreement for positive responses because low frequency of positive results can affect the total agreement percentage. Furthermore, agreement of detection results between two analyzers was determined by Cohen's kappa analysis [19]. Finally, the presence of propensity toward positive results in specific assay for certain allergen was determined when the difference between discrepant results accounted for over 10% of all pairs. For example, when assay A and assay B are compared for allergen C, [(number of samples with A positive, B negative result)−(number of samples with A negative, B positive result)]×100/ total number of results ≥10% can be interpreted as the positive propensity of assay A for allergen C compared to assay B.

For all statistical analyses, we used MedCalc 11.0 (MedCalc Software, Belgium) and SPSS 18.0 (SPSS Inc., Chicago, IL).

## 3 Results

### 3.1 Characteristics of study participants and paired sets

The serum samples from a total of 53 and 104 patients were tested for food panel and inhalant panel in this study. Characteristics of study participants are summarized in Table 2. Although several patients presented multiple allergic symptoms, urticaria was the most common clinical feature for participants in food panel while allergic rhinitis was the most frequent clinical symptoms for participants in inhalant panel. As mentioned earlier, different numbers of matched pairs were compared in each comparison analysis among four analyzers.

**Table 2:** Characteristics of study participants.

	Food panel	Inhalant panel
Total number	53	104
Demographic characteristics		
Number of male (%)	21 (39.6)	56 (53.8)
Age, median [1Q, 3Q]	32 [9,55]	35 [20.8, 56.5]
Age, range	1–85	5–78
Number of pediatric patients (%)^a^	16 (30.2)	24 (23.1)
Clinical symptoms and signs		
Urticaria (%)	32 (60.4)	3 (2.9)
Dermatitis (%)	14 (26.4)	1 (1.0)
Allergic rhinitis (%)	5 (9.4)	87 (83.7)
Allergic bronchitis (%)	1 (1.9)	4 (3.8)
Asthma (%)	2 (3.8)	5 (4.8)
Anaphylaxis or angioedema (%)	6 (11.3)	1 (1.0)
Others (%)	8 (15.1)^b^	4 (3.8)^c^
Matched pairs in each comparison		
AlloScreen vs. Allostation Smart II	43	90
Allostation Smart II vs. RIDA	30	79
Allergy-Q vs. RIDA	40	93

a Pediatric patients are defined as individuals with age <20 years.

b Others include xerotic eczema, erythema multiforme, drug eruption, and insect bite.

c Others include adenoid vegetation, chronic sinusitis, and sleep apnea.

### 3.2 Comparison between AdvanSure AlloScreen and AdvanSure Allostation Smart II for evaluation of upgrade

When we compared qualitative results between AdvanSure AlloScreen and AdvanSure Allostation Smart II, we used class 2 as the cut-off level for positive result since the manufacturer suggested the possibility of class 1 result indicating insufficient clinical significance to trigger allergic progression. A total of 43 and 90 paired serum samples were tested for 39 and 41 allergens in food and inhalant panel, respectively (Table 3). All allergens showed total agreement percentages over 93.0% and 92.2% in food and inhalant panel, respectively, which indicates good concordance between old and new versions of AdvanSure assays. However, 6 allergens (*Candida albicans*, cheddar cheese, chicken, *Cladosporium herbarum*, pork, yeast) in food panel and 4 allergens (dog, egg white, mackerel, soy bean) in inhalant panel showed no concordant positive result, possibly due to rare frequency of specific IgE antibodies to these allergens among Koreans and restricted number of paired samples in this study. On the contrary, two most common allergens in both food and inhalant panels which were *Dermatophagoides pteronyssinus* and *Dermatophagoides farina* showed high total agreement percentages of over 95.0% and high agreement levels with kappa indices over 0.9. However, total agreement percentage and kappa index decreased to 93.0% and 0.8, respectively, for house dust, which was the third most common allergen.

**Table 3:** Comparison between AdvanSure AlloScreen and upgraded AdvanSure Allostation Smart II using cutoff value of class 2.

Allergen	Food panel				Inhalant panel			
	*N*=43				*N*=90			
	Agreement (%)	Kappa index	Kappa index (95% CI)	Concordant positive rate (%)	Agreement (%)	Kappa index	Kappa index (95% CI)	Concordant positive rate (%)
Acacia					100.0	1.00	(1.00 to 1.00)	1.1
*Alternaria alternata*	100.0	1.00	(1.00 to 1.00)	11.6	100.0	1.00	(1.00 to 1.00)	3.3
Ash mix					100.0	1.00	(1.00 to 1.00)	1.1
*Aspergillus fumigatus*					100.0	1.00	(1.00 to 1.00)	1.1
Bermuda grass					97.8	0.74	(0.39 to 1.08)	3.3
Barley meal	97.7	0.66	(0.03 to 1.28)	2.3				
Beef	100.0	1.00	(1.00 to 1.00)	2.3				
Birch-Alder mix	97.7	0.79	(0.39 to 1.19)	4.7	96.7	0.38	(−0.17 to 0.93)	1.1
Buck-wheat	100.0	1.00	(1.00 to 1.00)	2.3				
*Candida albicans*	100.0	NA		0.0				
Cat	95.3	0.64	(0.18 to 1.10)	4.7	92.2	0.50	(0.18 to 0.81)	4.4
Cheddar cheese	100.0	NA		0.0				
Chicken	100.0	NA		0.0				
Citrus mix	100.0	1.00	(1.00 to 1.00)	2.3				
*Cladosporium herbarum*	97.7	0.00	(0.00 to 0.00)	0.0	98.9	0.66	(0.04 to 1.28)	1.1
Cockroach	97.7	0.79	(0.39 to 1.19)	4.7	97.8	0.74	(0.39 to 1.09)	3.3
Codfish	100.0	1.00	(1.00 to 1.00)	2.3				
Crab	93.0	0.54	(0.10 to 0.99)	4.7	96.7	0.39	(−0.15 to 0.93)	1.1
*D. farinae*	95.3	0.91	(0.78 to 1.03)	48.8	100.0	1.00	(1.00 to 1.00)	35.6
*D. pteronyssinus*	100.0	1.00	(1.00 to 1.00)	51.2	95.6	0.91	(0.81 to 1.00)	35.6
Dandelion					98.9	0.66	(0.04 to 1.28)	1.1
Dog	93.0	0.38	(−0.16 to 0.91)	2.3	97.8	−0.01	(−0.03 to 0.00)	0.0
Egg white	100.0	1.00	(1.00 to 1.00)	2.3	100.0	NA		0.0
Garlic	97.7	0.79	(0.39 to 1.19)	4.7				
Goldenrod					98.9	0.66	(0.04 to 1.28)	1.1
Hazelnut					98.9	0.66	(0.04 to 1.28)	1.1
House dust	93.0	0.82	(0.63 to 1.01)	23.3	93.3	0.84	(0.72 to 0.96)	26.7
Japanese cedar					98.9	0.66	(0.04 to 1.28)	1.1
Japanese Hop	100.0	1.00	(1.00 to 1.00)	2.3	98.9	0.66	(0.04 to 1.28)	1.1
Mackerel	97.7	0.66	(0.03 to 1.28)	2.3	100.0	NA		0.0
Milk	100.0	1.00	(1.00 to 1.00)	2.3	96.7	0.81	(0.59 to 1.02)	7.8
Mugwort	95.3	0.48	(−0.14 to 1.10)	2.3	100.0	1.00	(1.00 to 1.00)	1.1
Oak white	97.7	0.66	(0.03 to 1.28)	2.3	98.9	0.66	(0.04 to 1.28)	1.1
Onion	100.0	1.00	(1.00 to 1.00)	2.3				
Orchard grass					96.7	0.56	(0.12 to 1.00)	2.2
Oxeye daisy					98.9	0.66	(0.04 to 1.28)	1.1
Peach	97.7	0.66	(0.03 to 1.28)	2.3	97.8	0.49	(−0.11 to 1.09)	1.1
Peanut	100.0	1.00	(1.00 to 1.00)	2.3				
*Penicillium notatum*					100.0	1.00	(1.00 to 1.00)	2.2
Pigweed					100.0	1.00	(1.00 to 1.00)	2.2
Pine					100.0	1.00	(1.00 to 1.00)	1.1
Poplar mix					100.0	1.00	(1.00 to 1.00)	1.1
Pork	95.3	−0.02	(−0.06 to 0.01)	0.0				
Ragweed	97.7	0.79	(0.39 to 1.19)	4.7	94.4	0.26	(−0.18 to 0.70)	1.1
Reed					97.8	0.79	(0.51 to 1.07)	4.4
Rice	97.7	0.66	(0.03 to 1.28)	2.3				
Russian thistle					96.7	0.65	(0.29 to 1.01)	3.3
Rye pollens	97.7	0.79	(0.39 to 1.19)	4.7	95.6	0.65	(0.33 to 0.96)	4.4
Salmon	100.0	1.00	(1.00 to 1.00)	2.3				
Sallow willow					94.4	0.27	(−0.15 to 0.70)	1.1
Shrimp	93.0	0.54	(0.10 to 0.99)	4.7	95.6	0.32	(−0.16 to 0.80)	1.1
Soy bean	100.0	1.00	(1.00 to 1.00)	2.3	98.9	0.00	(0.00 to 0.00)	0.0
Sweet vernal grass					97.8	0.82	(0.58 to 1.06)	5.6
Sycamore mix					98.9	0.66	(0.04 to 1.28)	1.1
Timothy grass					97.8	0.79	(0.51 to 1.07)	4.4
Tomato	100.0	1.00	(1.00 to 1.00)	2.3				
Tuna	100.0	NA		0.0				
Wheat flour	97.7	0.79	(0.39 to 1.19)	4.7				
Yeast, bakers	100.0	NA		0.0				

NA: Not available.

### 3.3 Comparison of AdvanSure Allostation Smart II or PROTIA Allergy-Q with RIDA Allergy Screen applying cut-off level of class 2

We evaluated concordance rate of two newly developed fully automated assays (i.e. AdvanSure Allostation Smart II and PROTIA Allergy-Q) with results by RIDA Allergy Screen considered as the reference values in this study utilizing class 2 for the cut-off level for positive result (Table 4). Total agreement percentages were over 90.0% in most allergens in both assays for food and inhalant panels. However, allergens with the most frequent positive results (i.e. *D. farina*, *D. pteronyssinus*, house dust, and storage mite) presented concordance rates ranging from 69.6% to 90.0% for both AdvanSure Allostation Smart II and PROTIA Allergy-Q in food panel as well as inhalant panel.

**Table 4:** Comparison of two new fully automated analyzers with RIDA Allergy Screen as a reference using cutoff level of class 2.

Allergen	AdvanSure Smart II vs. RIDA				PROTIA Allergy-Q vs. RIDA			
	Food panel		Inhalant panel		Food panel		Inhalant panel	
	*N*=30		*N*=79		*N*=40		*N*=93	
	Agreement (%)	Concordant positive rate (%)	Agreement (%)	Concordant positive rate (%)	Agreement (%)	Concordant positive rate (%)	Agreement (%)	Concordant positive rate (%)
Acacia			100.0	1.3			97.8	0.0
*Alternaria alternata*	100.0	16.7	93.7	1.3	100.0	12.5	93.5	0.0
Anchovy	100.0	0.0						
Ash mix			100.0	1.3			96.8	0.0
*Aspergillus fumigatus*	93.3	0.0	97.5	0.0			97.8	0.0
Banana	100.0	0.0						
Barley meal	96.7	0.0			95.0	0.0		
Beef	96.7	0.0			97.5	0.0		
Bermuda grass			98.7	1.3			96.8	1.1
Birch-Alder mix	96.7	3.3	96.2	0.0	97.5	0.0	92.5	2.2
Bromelain (CCD)	93.3	0.0	92.4	1.3				
Buck-wheat	100.0	0.0			95.0	0.0		
*Candida albicans*	100.0	0.0			100.0	0.0		
Cat	96.7	0.0	88.6	2.5	97.5	0.0	93.5	2.2
Cheddar cheese	100.0	0.0			100.0	0.0		
Chestnut	96.7	0.0			100.0	0.0		
Chicken	100.0	0.0			100.0	0.0		
Citrus mix	100.0	0.0			97.5	0.0		
*Cladosporium herbarum*	100.0	0.0	96.2	0.0			97.8	0.0
Clam	100.0	0.0			100.0	0.0		
Cockroach	96.7	3.3	97.5	2.5	92.5	5.0	95.7	2.2
Codfish	96.7	0.0			100.0	0.0		
Crab	93.3	0.0	100.0	0.0	85.0	0.0	94.6	0.0
Cucumber	100.0	0.0						
*D. farinae*	76.7	26.7	77.2	12.7	76.7	30.0	82.8	11.8
*D. pteronyssinus*	80.0	26.7	75.9	13.9	90.0	22.5	89.2	12.9
Dandelion			100.0	1.3			96.8	2.2
Dog	96.7	0.0	97.5	0.0	87.5	5.0	97.8	2.2
Egg white	100.0	3.3	98.7	0.0	100.0	7.5	97.8	0.0
Garlic	100.0	0.0			95.0	0.0		
Goldenrod			97.5	1.3			95.7	0.0
Hazelnut			100.0	1.3			98.9	1.1
House dust	73.3	0.0	69.6	0.0			78.5	0.0
Japanese cedar			98.7	0.0			100.0	1.1
Japanese Hop	100.0	0.0	98.7	1.3	100.0	0.0	96.8	0.0
Kiwi	100.0	0.0			100.0	0.0		
Latex			98.7	0.0			96.8	0.0
Mackerel	96.7	0.0	100.0	0.0	95.0	0.0	98.9	0.0
Mango	100.0	0.0						
Milk	100.0	0.0	86.1	2.5	100.0	0.0	93.5	0.0
Mugwort	96.7	0.0	98.7	0.0	100.0	0.0	98.9	1.1
Mussel	100.0	0.0						
Oak white	100.0	3.3	98.7	0.0	100.0	2.5	96.8	0.0
Onion	100.0	0.0						
Orchard grass			100.0	1.3			97.8	1.1
Oxeye daisy			100.0	1.3			100.0	2.2
Peach	96.7	0.0	97.5	1.3	100.0	0.0	97.8	0.0
*Penicillium notatum*			96.2	0.0			97.8	0.0
Pigweed			100.0	1.3			98.9	1.1
Pine			98.7	0.0			98.9	0.0
Poplar mix			98.7	0.0			97.8	1.1
Peanut	100.0	0.0			100.0	0.0		
Pork	96.7	0.0			95.0	0.0		
Potato	100.0	0.0	97.5	0.0				
Pupa, silk cocoon	90.0	0.0			90.0	0.0		
Rabbit			98.7	0.0				
Ragweed	93.3	0.0	100.0	2.5	97.5	0.0	98.9	2.2
Redtop, bent grass			100.0	1.3				
Reed			96.2	0.0			97.8	0.0
Rice	100.0	0.0			95.0	0.0		
Russian thistle			98.7	1.3			97.8	1.1
Rye pollens	100.0	0.0	93.7	2.5	95.0	0.0	98.9	2.2
Sallow willow			100.0	1.3			97.8	2.2
Salmon	96.7	0.0			100.0	0.0		
Shrimp	96.7	6.7	94.9	0.0	95.0	0.0	100.0	0.0
Soy bean	100.0	0.0	100.0	0.0	100.0	0.0	100.0	0.0
Storage mite	86.7	6.7	89.9	10.1			90.3	11.8
Sweet vernal grass			94.9	1.3				
Sycamore mix			100.0	1.3			100.0	2.2
Timothy grass			94.9	1.3			97.8	2.2
Tomato	100.0	0.0			97.5	0.0		
Tuna	100.0	0.0			100.0	0.0		
Wheat flour	93.3	0.0			90.0	0.0		
Yeast, bakers	100.0	0.0			100.0	0.0		
Yellow jacket (wasp)			96.2	0.0				

Furthermore, several allergens which showed propensity toward positive result in specific assay were noticed in both comparison analyses (Table 5). While AdvanSure Allostation Smart II and PROTIA Allergy-Q showed positive propensity for some allergens when compared with RIDA Allergy Screen, RIDA Allergy Screen did not show positive propensity for any allergens with 10% discrepant results. For evaluation of AdvanSure Allostation Smart II, three allergens with the highest positive propensity results were *D. farina* (23.3%, 22.8%), *D. pteronyssinus* (20.0%, 24.1%), and house dust (26.7%, 30.4%) in both food and inhalant panels. Similarly three highest positive propensity results for PROTIA Allergy-Q were observed in *D. farina* (17.2%), *D. pteronyssinus* (10.8%), and house dust (19.4%) in inhalant panel. However, *D. farina* (17.5%) and dog (12.5%) showed highest positive propensity results in food panel of PROTIA Allergy-Q. Interestingly, the allergen with largest class difference between AdvanSure Allostation Smart II or PROTIA Allergy-Q and RIDA Allergy Screen was pupa silk cocoon in food panel although it showed positive propensity of 10.0% (i.e. mean difference by class 3 in AdvanSure Allostation Smart II vs. RIDA Allergy Screen comparison and mean difference by class 5 in PROTIA Allergy-Q vs. RIDA Allergy Screen comparison).

**Table 5:** List of allergens which present positive propensity in each assay.

Allergen	AdvanSure SmartII vs. RIDA		PROTIA Allergy-Q vs. RIDA	
	AdvanSure SmartII positive propensity (%)	RIDA positive propensity (%)	PROTIA Allergy-Q positive propensity (%)	RIDA positive propensity (%)
Food panel				
Cockroach	NS	NS	7.5	NS
* D. farinae*	23.3	NS	17.5	NS
* D. pteronyssinus*	20.0	NS	10.0	NS
Dog	NS	NS	12.5	NS
House dust	26.7	NS	NS	NS
Pupa, silk cocoon	10.0	NS	10.0	NS
Storage mite	13.3	NS	NS	NS
Wheat flour	NS	NS	10.0	NS
Inhalant panel				
Birch-alder mix	NS	NS	7.5	NS
Cat	8.9	NS	NS	NS
Crab	NS	NS	5.4	NS
* D. farinae*	22.8	NS	17.2	NS
* D. pteronyssinus*	24.1	NS	10.8	NS
House dust	30.4	NS	19.4	NS
Milk	6.3	NS	NS	6.5
Storage mite	7.6	NS	NS	NS

NS: Not significant, defined as discrepant results less than 5%.

### 3.4 Effects of lowering or raising up the cut-off level for positive result

**Fig. 1:**
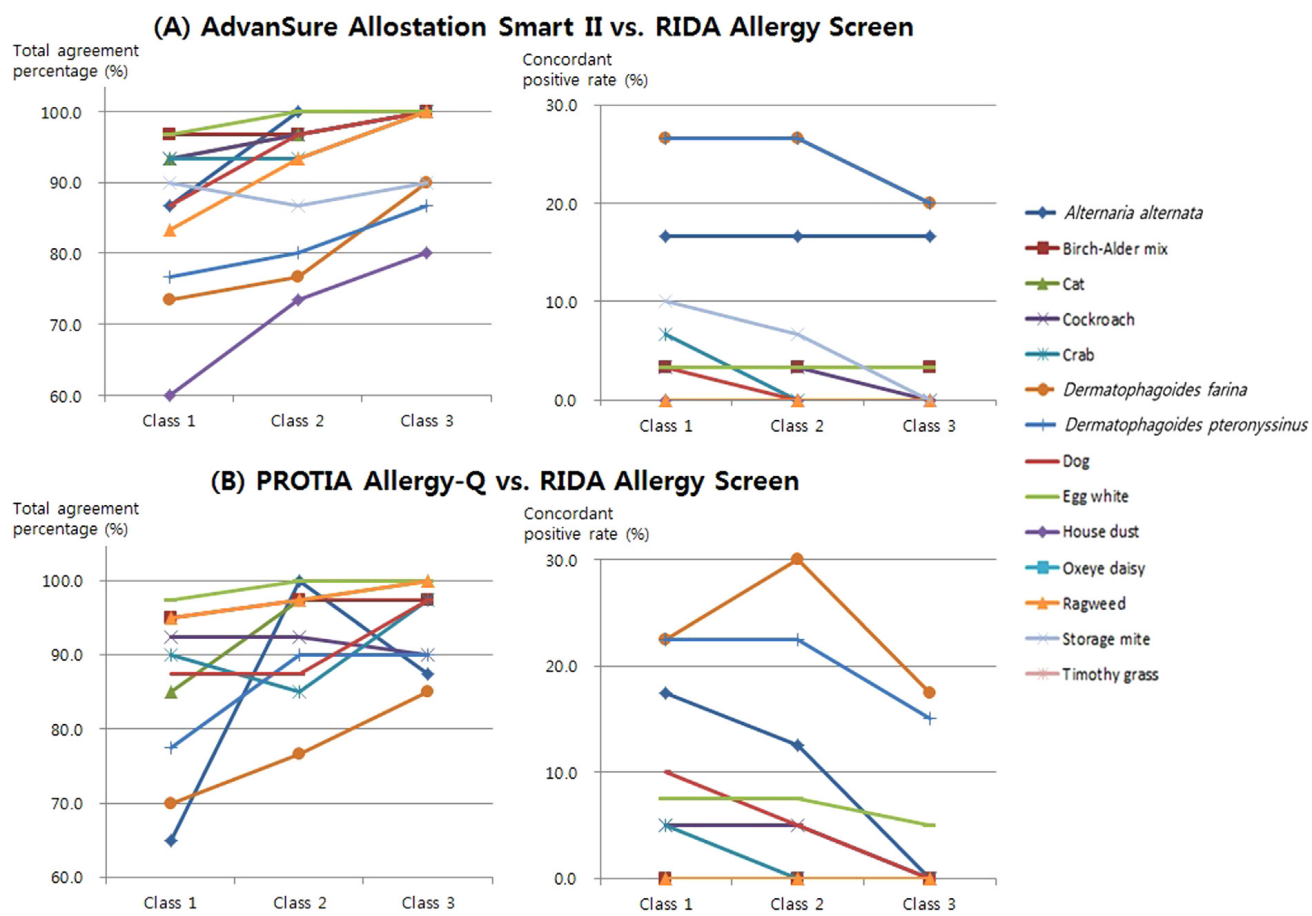
Changes of total agreement percentage and concordant positive rate according to three different cut-off levels for positive result determination in the food panels.

**Fig. 2:**
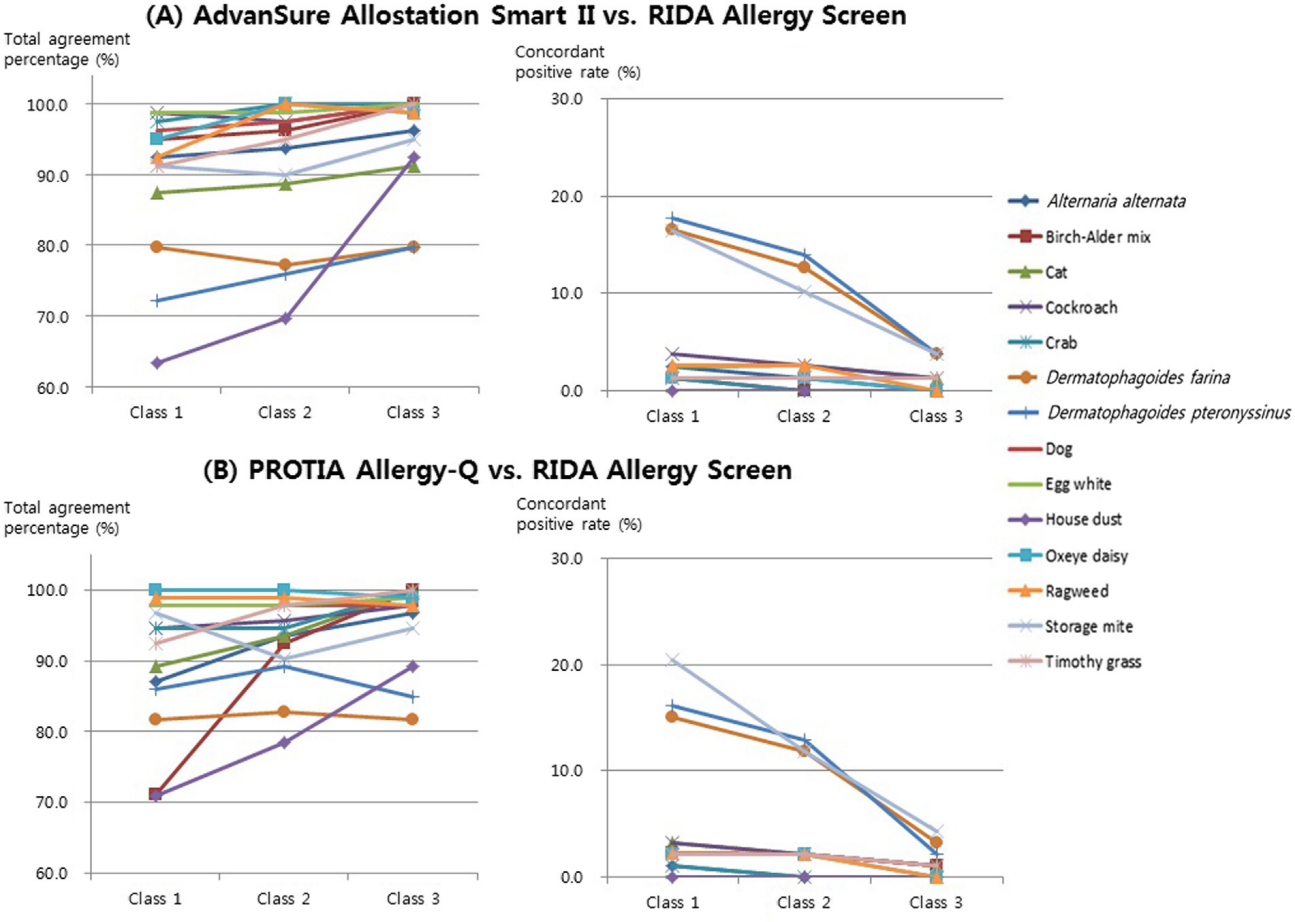
Changes of total agreement percentage and concordant positive rate according to three different cut-off levels for positive result determination in the inhalant panels.

To evaluate the effects of various cut-off levels for positive result determination, we applied two more cut-off levels other than the conventional criteria of class 2 as minimal requirement for positive result; class 1 and class 3 as cut-off levels. Total agreement percentages and concordant positive rates were fairly influenced by application of both higher and lower cut-off levels (Fig. 1, Fig. 2). Since higher cut-off level led to more negative results, concordant positive rates decreased naturally. However, the changes of total agreement percentage according to the increase in cut-off level varied among allergens by different analyzers.

### 3.5 Evaluation of positive rates for unique antigens in specific analyzer

Since different analyzers include various allergens, analyzer-specific allergens present diverse frequencies among patients (Table 6). Among the accretional allergens introduced in Advansure Allostation Smart II, *Acarus siro* and apple in inhalant panel showed significant positive rates of 20.0% and 8.9%, respectively, when class 1 was utilized as cut-off level. When cut-off level was increased to class 2, these positive rates decreased to 12.2% and 4.4%, respectively (data not shown).

**Table 6:** List of analyzer-specific allergens and frequencies of positive results using cut-off level of class 1.

Food panel						Inhalant panel					
AdvanSure Smart II		PROTIA Allergy-Q		RIDA		AdvanSure Smart II		PROTIA Allergy-Q		RIDA	
*N*=40		*N*=40		*N*=40		*N*=90		*N*=93		*N*=93	
Allergen	Positivity frequency (rate, %)	Allergen	Positivity frequency (rate, %)	Allergen	Positivity frequency (rate, %)	Allergen	Positivity frequency (rate, %)	Allergen	Positivity frequency (rate, %)	Allergen	Positivity frequency (rate, %)
Alder	2 (5.0)	Almond	2 (5.0)	Chocolate	0	*Acarus siro*	18 (20.0)	Weat flour	3 (3.2)	Anchovy	0
Celery	1 (2.5)			Latex	0	Alder	2 (2.2)			Banana	0
Cacao	0			Lilac	0	Apple	8 (8.9)			Chestnut	1 (1.1)
Maize	1 (2.5)			Redtop, bent grass	0	Cacao	0			Chocolate	0
Mushroom	0			Wool	0	English plantain	1 (1.1)			Clam	0
Sesame	1 (2.5)			Yellow jacket (wasp)	1 (2.5)	Guinea pig	0			Cucumber	2 (2.2)
Squid	2 (5.0)					Hamster	0			Kiwi	1 (1.1)
						Hinoki cypress	1 (1.1)			Lilac	1 (1.1)
						Honey bee	3 (3.3)			Mango	0
						Horse	3 (3.3)			Mussel	0
						Maize	1 (1.1)			Wool	0
						Sesame	1 (1.1)				
						Sheep	1 (1.1)				

### 3.6 Frequency of multiple allergen positive results per patient by four different analyzers

Because multiple allergen positive results might indicate cross-reactivity between similar allergens, frequency of patients with two or more positive results was analyzed according to four different analyzers. By application of class 2 as the cut-off level for positive result, AdvanSure AlloScreen and AdvanSure Allostation Smart II presented highest frequency of patients with multiple positive allergens (i.e. AdvanSure AlloScreen: *N*=30 for food panel and *N*=43 for inhalant panel, AdvanSure Allostation Smart II: *N*=23 for food panel and *N*=37 for inhalant panel) with maximum positive allergen numbers of 23 and 34 in food panel and 28 and 32 in inhalant panel, respectively.

## 4 Discussion

During the last decade, there have been several remarkable introductions of new MAST assays by different manufacturers into the clinical field of allergic diseases. Accordingly, evaluation and comparison studies of these novel MAST analyzers were reported by few groups [6], [8]. Until today, a total of four MAST assays [i.e. AdvanSure AlloScreen, RIDA Allergy Screen, MAST Optigen (Hitachi), and Polycheck (Biocheck)] were frequently evaluated with each other and showed comparable clinical performances [10], [11], [20]. Recently, Lee and colleagues presented favorable performance of newly developed PROTIA Allergy-Q [12]. In this current trend, we evaluated four MAST analyzers including two newly developed and fully automated assays. This study is the first evaluation report for AdvanSure Allostation Smart II and only the second comparison study for PROTIA Allergy-Q. Also, our study is unique for evaluating upgraded version of specific assay to ensure the improvement by including both AdvanSure AlloScreen and AdvanSure Allostation Smart II.

Based on our results, most results of comparison analyses presented good concordance levels by means of total agreement percentages over 90.0%. Satisfactory agreements were observed not only in the comparison between AdvanSure AlloScreen and AdvanSure Allostation Smart II, but also in the evaluation of AdvanSure Allostation Smart II and PROTIA Allergy-Q compared with RIDA Allergy Screen. Although four allergens with the most frequent positive results, which were *D. farina*, *D. pteronyssinus*, house dust, and storage mite, showed slightly lower concordance rates, these different results could be sufficiently overcome by careful interpretation of MAST results in association with clinical manifestations.

One interesting finding we focused on in this study was positive propensity of each analyzer. In the midst of various available MAST analyzers with comparable diagnostic performance, it is important for laboratory physicians to recognize the unique propensity of each analyzer which might easily lead to positive results for particular allergen. Our study suggests that AdvanSure Allostation Smart II and PROTIA Allergy-Q are more sensitive or prone to report positive results for three common allergens (i.e. *D. farina*, *D. pteronyssinus*, and house dust) in both food and inhalant panels than RIDA Allergy Screen. Considering the multiple positive result frequencies related with cross-reactivity among similar allergens as possible mechanism for explanation [21], [22], [23], positive propensity of each analyzer should be cautiously understood. Moreover, variations in the allergen extraction method by different manufacturers might have caused this phenomenon of diverse positive propensity in each analyzer.

Adding new allergens in the panel list is another issue for future development of MAST analyzers. Candidate allergens should be assessed based on evidences for continuous and dramatic changes in the environment and socio-behavioral lifestyle of modern individuals [24], [25]. At the same time, cost-effective approach is required for choices of clinically efficient allergen with reference to epidemiologic results of geographically characteristic allergen studies [26], [27], [28], [29]. Our results support the significant positive rate for *Acarus siro* among Korean population [30], which is unique allergen included only in Advansure Allostation Smart II inhalant panel. Further investigations for *Acarus siro* as an inhalant allergen in general population might highlight the advantage of Advansure Allostation Smart II.

One of the most important approaches we performed in this study was the re-evaluation of cut-off levels in order to avoid false positive results. Besides the conventional cut-off level of class 2 as the minimal positive result criteria, we analyzed the changes of total agreement percentages and concordant positive rates according to cut-off level decrease to class 1 or increase to class 3. Although the increase of cut-off level seemed to make clinical circumstance more simple and concise by presenting only the definitive positive allergens, this modification resulted in lower concordant positive rates with possibility of missing potentially critical allergens. On the other hand, decrease of cut-off level produced lower total agreement percentages in most allergens, which might obscure physicians from clear identification of clinically relevant allergens. Detailed evaluation with similar approach for optimal cut-off class should be conducted for each analyzer according to specific regional frequency and distribution of allergens in the future.

A critical limitation of this study was the use of RIDA Allergy Screen assay as the reference value for evaluation of newly developed analyzers. Among the comparison studies published until today, most studies included the ImmunoCAP system (Phadia, Uppsala, Sweden) for comparison analyses as an empirically reference method [8], [10], [11], [12], [20]. However, the ImmunoCAP system is neither the official nor the definite reference procedure for measurement of allergen-specific IgE antibodies despite its good reliability and reproducibility. While the ImmunoCAP system might be impractically expensive for efficient clinical service for small to medium sized clinical laboratories [31], RIDA Allergy Screen assay has been continuously evaluated and reported for favorable clinical correlation with not only the ImmunoCAP system, but also serum total IgE [10], [32]. We anticipated that objective comparison between currently available MAST analyzers might provide sufficient information for clinical use in the practical medical field.

In conclusion, AdvanSure Allostation Smart II maintained steady concordant performance in the upgrade process from AdvanSure AlloScreen, with the uniquely extended allergen list including *Acarus siro* which showed certain positive rates. AdvanSure Allostation Smart II and PROTIA Allergy-Q presented favorable agreement performances with RIDA Allergy Screen, although positive propensities were noticed in some allergens. The conventional cut-off level of class 2 as the minimal positive result criteria appeared to be suitable for current MAST analyzers in the clinical interpretation.

## Conflict of interest

All authors declare no conflict of interest.
